# Development of a Web-Based Training Platform for School Clinicians in Evidence-Based Practices for ADHD

**DOI:** 10.1007/s12310-022-09556-9

**Published:** 2022-11-23

**Authors:** Linda J. Pfiffner, Melissa R. Dvorsky, Lauren M. Friedman, Lauren M. Haack, Sara Chung, Julia M. Charalel, Elizabeth Hawkey, Madeline Spiess

**Affiliations:** 1 Department of Psychiatry and Behavioral Sciences, University of California San Francisco, 675 18th St, San Francisco, 94143 CA US; 2 Department of Pediatrics and Psychiatry, Children’s National Hospital, George Washington University School of Medicine, Washington DC, US; 3 Department of Psychology, Arizona State University, Tempe, AZ US

**Keywords:** Attention-deficit/hyperactivity disorder, Clinician training, Web-based training, Behavioral intervention, School–home intervention

## Abstract

Lack of training for school clinicians in evidence-based practices (EBPs) contributes to underutilization of such services for youth with attention-deficit/hyperactivity disorder (ADHD). Advances in web-based technology and videoconferencing have allowed for expanded access to and optimization of training. We describe the development and outcomes of a novel web-based platform for training school clinicians to gain skills in EBPs for school-age youth with ADHD. The training platform is adapted from an empirically supported, in-person training for a school–home behavioral intervention (Collaborative Life Skills program) and includes skill modules for working with teachers, parents, and students. Training methods include web-accessed manuals/handouts, skill example video clips, automated progress monitoring tools, and consultation/in-session coaching via videoconferencing. We gathered stakeholder qualitative and quantitative feedback during discovery and design phases of the iterative development. We then evaluated the usability, acceptability, fidelity and clinician and student outcomes of the remote training program. Focus group themes and qualitative feedback identified clinician preferences for remote training features (e.g., interactive, brief, role-plays/coaching methods), video tools (recorded samples of skills and therapy sessions), and progress monitoring tools (e.g., clear, easy to use). Clinician usability ratings of the platform were high with most components rated as moderately to very useful/easy to use. Clinician ratings of usability, fidelity implementing the treatment, and their EBP knowledge and confidence following training were favorable. Student’s outcomes were similar to those achieved in prior studies of clinician in-person training. Results support the promise of remote, web-based clinician training for the dissemination of evidence-based practices.

Attention-deficit/hyperactivity (ADHD)-related behaviors and impairments are among the most common reasons for referrals to school mental health providers (SMHPs; Harrison et al., 2012). Organization skills, time management, classwork and homework completion, test and report card grades, teacher ratings of behavior, and academic achievement are all significantly lower among youth with ADHD relative to those without the disorder (DuPaul & Langberg, 2015). Social impairments are common. Many children with ADHD have few friendships, and at least half of those diagnosed with ADHD are rejected by their peers, while many others are neglected or ignored by their peer group (Mikami, 2010). Family functioning is characterized by parent–child conflict, parental stress and high levels of negative/ineffective parenting (Johnston & Mash, 2001) which disrupt interpersonal relationships and cause significant distress for children with ADHD and their families (Kaiser et al., 2011). Youth with ADHD are disproportionately likely to experience special education placement, grade retention, school failure, and drop-out (Kuriyan et al., 2013), and as a result of these impairments, students with ADHD cost the US education system approximately $13.4 Billion USD annually (Robb et al., 2011).

Evidence-based psychosocial interventions (EBPs) for ADHD such as behavioral parent training, classroom behavior management, peer interventions, and organizational skills training, show clinically significant benefits for youth and their families (Evans et al., 2018). However, most of these interventions have been developed for clinic settings or for implementation by research staff, and few evidence-based practices reach children with ADHD within schools (Spiel et al., 2014). The typical individual and group-based counseling services provided in schools fail to apply strategies for generalizing treatment gains across settings and critically do not include the classroom and home-based interventions that are crucial components of EBPs for ADHD (Fabiano & Pyle, 2019). A contributing factor to the limited use of EBPs for ADHD in school settings is the lack of evidence-based training programs for providing these approaches. In order for professional training to have a pronounced, durable effect on clinician competence to implement and use EBPs, active learning strategies and ongoing coaching, supervision and feedback are needed (Beidas et al., 2012; Lyon et al., 2011; Owens et al., 2014; Rakovshik et al., 2016). While coaching is an essential component of effective training, it is one of the least available due to its resource demands. School staff also need training and consultation that can flexibly accommodate their busy school day. These factors constitute significant barriers to adoption of EBPs within most school systems (Stewart et al., 2012).

To address this gap, we developed the Collaborative Life Skills Program (CLS) which provides much needed EBP services to students while concomitantly training SMHPs on delivering the intervention using a holistic, multimodal approach. CLS combines three evidence-based behavioral treatments all adapted for school-based delivery: behavioral parent training, daily report card (DRC) with teacher consultation, and child skills training (Pfiffner et al., 2016). This combination of interventions was designed to address each of the common impairment domains for youth with ADHD (academic, social, family). SMHPs lead the intervention components at school. *Parents* attend group meetings focused on promoting parent behavior management strategies at home (e.g., ABC model, praise, rewards, effective instructions, routines, planned ignoring, response cost, parent stress management). *Teachers* implement a customized school–home DRC and evidence-based strategies to scaffold and support attention, behavior, and motivation in the classroom (Fabiano et al., 2010; Pfiffner, 2011). *Students* attend group sessions focused on building independence, organization, and social skills (Pfiffner & McBurnett, 1997). Parents and teachers are taught to reinforce skills taught to children for generalization and to specifically promote motivation, behavioral regulation, and organizational deficits. Delivery is coordinated such that parents, students, and teachers are simultaneously trained in their aspects of the treatment using the same terminology. Reinforcement contingencies are set within and across settings, resulting in around-the-clock support of student behavior across impairment domains and settings.

The training program for school clinicians in delivery of CLS incorporates key elements of empirically supported training approaches and adult learning, including highly relevant exemplars with video examples, active learning techniques, spaced learning sessions (weekly) with material taught just prior to implementation, synchronous practice sessions with other trainees, and on-site, in vivo coaching and performance feedback to promote accurate application and accountability (Herschell et al., 2010). The training program includes a day-long introductory workshop, weekly consultation sessions, live observation and coaching from a study clinician trainer during each SMHP-lead parent, student, and teacher group session, and immediate performance feedback after each session. These methods were successful in achieving high SMHP implementation fidelity and acceptability in several trials (Pfiffner et al., 2011, 2016). Multiple iterations of the program across schools showed significant between-group gains (most with medium to large effect sizes) for CLS relative to usual school services on ADHD and ODD symptoms and impairment, and most parent-reported gains were maintained in the subsequent school year (Pfiffner et al., 2016, 2018).

Despite these promising results, the CLS training approach was limited in the following ways: the on-site coaching and feedback components of the training were time intensive and costly. The associated high travel expenses and scheduling demands (including work time lost for travel time for trainers and trainees) greatly limited scalability, making widespread dissemination within and outside of the immediate geographic area prohibitively expensive. In addition, CLS training and group materials were not portable or easily accessible, impeding sustainability of program implementation.

To address these limitations and broaden the reach of EBPs for schools, SMHPs, and students, we turned to web-based, remote training models. Improvements in technology have made web-based training, coaching, and supervision a viable, effective, engaging, and accessible alternative to in-person training (Khanna & Kendall, 2015; Rakovshik et al., 2016). Although the COVID-19 pandemic-related school closures in March, 2020, dramatically increased the use of telehealth for clinician training and direct service (Torous et al., 2020), the need for remote training to address the geographic distance, cost and scalability barriers of in-person training and the shortage of clinicians trained in EBPs was apparent before the pandemic. A growing body of research supports the acceptability, feasibility, and effectiveness of web-based training for mental health providers (Khanna & Kendall, 2015). For example, the combination of web-based self-guided material and live remote coaching/feedback during role plays with mock patients using video teleconferencing from an expert therapist has been shown to have high user satisfaction and adherence and to improve knowledge and clinician trainee skills for implementing CBT for anxiety (Kobak et al., 2013, 2017) and BPT for disruptive behavior (Ortiz et al., 2020)*.* Consistent with outcomes from in-person workshops, when online trainings *do not* incorporate remote coaching or consultation while treatment is being delivered the knowledge acquired through web-based training does not translate into gains in clinical proficiency or use of EBPs (Beidas et al., 2012; Frank et al., 2020; Herschell et al., 2010; Rakovshik et al., 2016). This suggests that coaching or consultation is necessary to support clinician uptake of EBPs regardless of whether training occurs online or face-to-face and may be especially important for complex EBTs with several or more components (Frank et al., 2020). To our knowledge, there are no published feasibility studies of web-based remote training for school mental health providers in behavioral interventions for ADHD.

As a first step in translating our in-person training to a remote format, we conducted a feasibility study of a remote version of CLS (CLS-Remote or CLS-R) for training school clinicians in evidence-based practices for attention and behavior problems. CLS-R is based on CLS with one major modification: CLS-R utilizes web-based technology to provide remote training, consultation, and supervision while still adhering to principles of adult learning and empirically supported training methods that guided our original in-person training approach. Goals of the feasibility study reported herein were to: 1) develop the remote training program for school clinicians by applying a user-centered design (Lyon et al., 2019), in the context of interactive discovery, design, build, and test phases and 2) evaluate multiple feasibility dimensions of the remote training based on input from key stakeholders (SMHPs, teachers, parents). Drawing from feasibility frameworks outlined by (Gadke et al., 2021; Lyon et al., 2019; Proctor et al., 2011), we prioritized evaluation of the following four dimensions of feasibility most relevant for guiding development of the new remote training methods: usability (degree to which training/intervention can be used easily, efficiently, and with satisfaction/low user burden by a particular stakeholder), acceptability (perception of training/intervention as appropriate, palatable, satisfactory), implementation (fidelity of training/intervention implementation), and effectiveness (training/intervention outcomes). Findings are intended to inform future large-scale controlled evaluations of the remote training program.

## Methods

### School Partnerships and Design

We collaborated with an urban school district in Northern California serving over 57,000 students to develop and implement CLS-R with SMHPs at district K-5 elementary schools. Participating schools averaged 409 students (range: 216–547), with 40.6% of students who qualified for free or reduced lunch (range: 21–89%). We conducted initial stakeholder focus groups (2017–2018); open trials of CLS-R (2018–2019); and a pilot RCT of CLS-R vs. CLS (2019–2020), the latter phase terminated prematurely due to COVID-19 school closures and we only include the CLS-R schools that had completed the study prior to the closures. Prior to participation, SMHPs reviewed institutional review board-approved study descriptions and signed consent forms.

## SMHP Characteristics

SMHPs were masters-level school social workers in local elementary schools who coordinated student support services, including behavior support planning, individual and group mental health services, and crisis counseling at their school. We recruited 8 SMHPs (one per school) through our school liaison, announcements in school district news blurbs, and direct outreach to SMHPs who had prior experience with CLS. Six of these were recruited for and participated in the discovery phase. Four of these six also participated in the test phase along with two additional SMHPs were recruited for and participated in the test but not the discovery phase. As a result, the perspectives of SMHPs who participated in the discovery phase were also included during the test phase. SMHPs, on average, worked and trained in the mental health field for 11.3 years (range: 4–19 years), in the district for 5.5 years (range: 3–11 years), and at their current school for 3.9 years (range: 0.25–9 years). All had experience leading skills-based child groups (average 16.8 groups each, range 6 to > 20 groups) but little experience leading behavioral parenting groups (specifically, 4 SMHPs had each led one group); all had consultation experience with teachers using a DRC (ranged from 5–10 cases to more than 20 cases). Four of the SMHPs (two participated in the discovery phase only and two participated in the discovery and test phases) had experience with a previous version of the CLS model approximately 5 years prior to participating in this study. However, SMHPs with and without prior CLS experience reported similar overall experience with behavioral interventions prior to CLS-R participation. SMHPs received extended calendar pay at a rate similar to their school salary for attending training sessions and participating in program development group meetings, which occurred outside of their salaried positions. They implemented the intervention sessions as part of their salaried positions.

## Trainers

Trainers included five clinical psychology PhDs (including the senior author and CLS developer) and one masters-level predoctoral intern. Trainers had considerable expertise in implementing behavioral EBPs with children with ADHD and related problems. Two had been trainers for the in-person version of CLS and trained the trainers new to the program (see Training Team section below).

## Program Development

We applied an iterative development process consisting of: Discovery, Design, Build and Test phases (Lyon et al., 2019). Our goal was to develop a remote option for each of the CLS components. See Table 1 comparing the in-person (CLS) and remote-training methods (CLS-R).

**Table 1:** CLS Training Components

Core Component	CLS	CLS-R
Manuals, Materials, Guidelines	Materials kept in large paper binders by SMHPs	Materials provided on *website* with 24 h access from any location
Initial Workshop & Weekly Group Consultation	Trainers/SMHPs travel to school sites to provide in-person training: one 6-h workshop; eight weekly 90-min consultations Learning strategies include didactic instruction, role plays, review of session videos, case review	Training provided via *videoconferencing*: three 2-h workshops; eight weekly 90-min consultations Learning strategies are adapted for videoconferencing, strategies target SMHP engagement during remote consultation, case review using progress graphs
Live session observation and coaching	Trainers travel to school sites to observe sessions: eight* 60-min parent sessions, eight 45 min child sessions, two 30–60 min teacher meetings, one to two 30 min teacher/parent/student meetings per student Real-time coaching provided by trainer during each session Immediate post-session feedback provided in-person	Sessions observed via *videoconferencing*: eight* 60-min parent sessions, eight 45 min child sessions, two 30–60 min teacher meetings, one to two 30 min teacher/ parent/student meetings per student Remote real-time in-session coaching provided remotely Post-session performance feedback provided via *videoconferencing*
Progress Monitoring	Paper measures used to evaluate weekly student progress During consultation SMHPs and trainers jointly review surveys qualitatively to guide troubleshooting/ tailoring for students/parents	*Online surveys* evaluate weekly student progress Student surveys scored/graphed automatically SMHPs and trainers jointly review graphed data remotely during consultation to guide troubleshooting/ tailoring for students
Fidelity Monitoring	Paper post-session fidelity ratings completed by trainers Training & feedback based on qualitative impressions	*Online surveys* of fidelity completed by trainers after each session Training & feedback are tailored based on trainer’s quantitative and qualitative evaluations

^*^The parent component, originally 9 group sessions, was reduced to 8 to match the number of child sessions and increase efficiency. The same topics were covered regardless of number of groups

### Discovery Phase

We conducted two 90-min focus groups with support from our web software developers to gather input from key stakeholders (SMHPs). The first focus group included 4 SMHPs who had prior experience with CLS, 2 software developers, and 2 CLS trainers. We included SMHPs with prior CLS experience in order to obtain feedback about remote training options from those who were familiar with our in-person training procedures. The second focus group included 2 SMHPs without prior experience in CLS, 2 software developers and 2 CLS trainers. Focus groups included queries about SMHP technology use, and preferences regarding web-based document and materials accessibility, strategies to maximize engagement during videoconference consultation, use of video tools to supplement training, mechanisms for providing feedback and coaching during live videoconference session observations, options for student progress monitoring, and remote methods to support EBP sustainability. Additional queries about active learning strategies for remote consultation were added to groups who were previously trained in CLS given their familiarity with the in-person procedures. We also queried about remote training frequency and length to assess feasibility. SMHPs were paid $50 for participation in the focus group.

Recorded focus group sessions were transcribed and summarized for themes related to the usability and feasibility of CLS-R. We applied content analysis to condense focus group data into a thematic framework. SMHP preferences voiced during focus groups revealed key themes for the CLS-R training components and features and included easily accessible intervention content, interactive and synchronous trainings, unobtrusive in-session coaching methods, and supplementary videos depicting parenting skill use. Example quotes are provided in Table 2.

**Table 2:** Key Themes from Initial SMHP Focus Groups

*Online training materials*
SMHPs highlighted the need for easy access to well-organized training materials with flexible options for electronic and downloadable materials with comments such as: *“I want to be able to watch videos and prepare for a meeting at home anytime”* *“I want the ability to access training materials, progress tools, videos and handouts, all from one place”* “*I want lessons and templates that can be printed out…I like to highlight by hand”* *“Takes too long to print and organize all the papers, so electronic would be better*
*Online training format*
Emphasized the importance of interactive, synchronous training sessions and stated that the session length should be shorter for remote than in-person training to optimize attention and engagement with comments such as: “*Online coursework is boring if not enough interaction to ensure you’re still engaged*” *“If there were other people engaging with me, I would be more accountable”* *“I absorb things [better] in conversation, than being lectured/read to”* *“8 h (length of in-person training sessions) is too long to stare at a computer and not feasible…2 h would be good.”* *“4-6 pm after school”*
*Remote Coaching Methods *
SMHPs stated that methods should not interfere or be distracting during session delivery and should maximize clear, efficient communication between trainer and SMHP with comments such as: “*Texting could be a problem due to unreliability, also appearance of rudeness to group members and distracting”* *“Earpiece overwhelming”* “*Having trainer communicate directly over zoom conferencing seems feasible”* *“Students would be open to someone on the screen”* SMHPs liked idea of saying *“let’s ask coach”* during parent and child groups and teacher sessions, or have planned pauses during the session to get coach input, but in either case they emphasized that the remote trainer should introduce themselves at the beginning of the session
*Supplementary Video Tools for Training*
SMHPs liked the idea of supplementary video tools but emphasized that they should be brief, have clear teaching points, and provide real-life examples of best practices for the intervention with comments such as: *“Videos should be quick and short”* “*Bulleted keywords or points, conversational language important, planned pauses where parents can ask questions, parents could view at home”* *“It’s best if it is real people”* *“Live actors would be amazing…to help parents understand [skills] that a lot miss”* *“Show me examples of best practices “* *“Would be helpful to show examples of [parenting strategies] that are working and not working”*
*Progress Monitoring Tools*
Tools to assess student progress should be easy to see and understand and accessible to all parties (i.e., SMHPs, parents, teachers) with comments such as: “*These should be easy to see and understand”* *“Prefer line graphs, be able to highlight progress”* *“Be able to show on laptop or mobile device for teachers* *“Parents and teachers should be able to view progress”*

### Design and Build Phases

Focus group data gathered during the discovery phase were reviewed with web-designers to create visual prototypes of CLS-R components and establish a prioritized list of features and content for SMHPs and trainers. The research team attended daily “scrum” videoconference calls (i.e., to address questions from the developers) and biweekly “sprint demos” (i.e., after building each set of features) with the developers to ensure co-development throughout the build phase. Revisions were made continually during this process based on CLS needs, feedback from SMHPs and trainers, and the capability of the web-platform. Based on the discovery phase results, we expanded the scope to include teacher and parent views to facilitate progress monitoring, sharing CLS materials, and an interactive calendar for scheduling meetings. The following elements/components were created during the build phase:

## CLS-R Website

The website was built using Salesforce Community Sites (Salesforce, 2018). This platform was selected given an ongoing collaboration between our university and Salesforce and the platform’s “out-of-the-box” capabilities which increased the feasibility of the build by allowing the research team to develop content and pages using button clicks and (i.e., without requiring coding experience). The website included customized portals for trainer, SMHP, teacher and parent users with layout designs intended to facilitate engagement and usability. All CLS manuals, session materials, troubleshooting guides, and training videos were embedded in the web portal. The SMHP view included: dashboard with tabs for their program materials (session outlines/scripts, session materials list, pdfs of session handouts, demonstration videos and handouts to be shared in parent and teacher sessions, and sample training videoclips), calendar for training and program sessions with direct links to videoconferencing rooms, and progress pages with graphs for each student’s DRC goals and parent/teacher ratings of global improvement. The trainer view included a dashboard with all materials available to SMHPs, plus trainer scripts that contained information to provide to the SMHPs and embedded handouts to facilitate screensharing during remote training sessions. Parent and teacher views included session handouts, video tools, and student progress pages. Based on trainer and SMHP feedback during test runs of website features during the build phase, we added an overview of the online tools to the first CLS training workshop and made a number of enhancements to the web-platform (e.g., enhanced formatting, infographics, streamlined links to surveys/materials, interactive troubleshooting guides, smart search tool, topic tagging, discussion boards).

## Training Team

Our research team trained three CLS-R cohorts (each consisting of two SMHPs trained in pairs) across the study period. Senior trainers (who were trainers in the original CLS trial) co-delivered the workshops and weekly consultations with junior trainers (those gaining experience training SMHPs). Each SMHP was assigned a primary senior trainer who, along with a junior trainer, conducted live session observation, coaching, and fidelity monitoring. The training team met weekly to discuss training questions, review content and troubleshoot issues.

## Videoconferencing for Training and Coaching

Synchronous videoconferencing (via Zoom) was embedded into the CLS-R site for the training workshops, weekly consultations, and live session observation and coaching. All SMHPs had district-provided laptops to use throughout the study.

## Workshops and Weekly Consultation

Session materials were adapted for videoconferencing format to allow trainers to privately view trainer scripts/prompts while screensharing the SMHP scripts, handouts, and videos. To promote engagement and interactive training opportunities, SMHPs were trained with one other SMHP from another participating school site. Trainers incorporated several training methods including frequent content questions, soliciting SMHP input and balanced participation, sample CLS delivery videoclips, and role play activities specifically designed for the remote environment. The timing, length, frequency, pacing, and content of training role play exercises, and number of video examples during training were adjusted based on SMHP feedback (e.g., full day in-person workshop divided into 3, 2-h remote sessions for CLS-R). The length of 8 weekly consultations (90 min) during the program remained the same as the original CLS in-person training. In total, SMHPs attended approximately 18 h of training workshop/consultation meetings. We iteratively refined videoconferencing features (e.g., tailored interactive activities; improved web layout to support streamlined screensharing).

## Live Session Observation and Coaching

During program sessions, SMHPs positioned their laptops to allow trainers to view and hear all student/parent/teacher participants and maintain direct view of the SMHP. Per SMHP feedback, live coaching prompts were delivered over Zoom verbally or visually (e.g., miming or directly prompting a behavior management strategy) by their primary trainers.

## Video Tools

Training also incorporated videoclips of best practices using peer exemplar recordings from previous trials for SMHPs with teaching points at the end of each video. Due to SMHP interest in accessing video recordings outside of training sessions, the videoclips were embedded next to session scripts on their portal which was accessible 24/7. In response to SMHPs preference for real-life examples of parents demonstrating specific parenting skills, we created scripts and recorded videos with actor parents and children demonstrating effective and ineffective parenting strategies to align with the curriculum. Videos were shown on a study-provided tablet or a school laptop during the parent group.

## DRC Tool and Progress Monitoring

Electronic tools to track student progress were adapted from printed materials and included weekly global improvement ratings completed by teachers and parents and DRC ratings. DRC target behavior goals are selected in the first collaborative parent–teacher meeting facilitated by the SMHP who then entered them into the students’ profile on the CLS-R website. The CLS-R website uses “smart text” to suggest sample target behavior goals from a bank of existing DRC targets and allows the user to write in their own goals. Once setup, the CLS-R website automatically sent teachers an electronic DRC to complete daily at specified times and results were immediately pushed to parents and linked back to the website where students’ data were extracted and graphed automatically for the SMHP, parent, teacher, and trainer views. Data from weekly progress monitoring measures were also graphed automatically and reviewed during weekly consultations to identify students in need of additional support, facilitate troubleshooting, and guide DRC modifications (e.g., when and how to change target behaviors) with teachers as students progressed in the program. Based on stakeholders’ feedback, we modified our progress monitoring tools to improve usability (e.g., automated prompts, enhanced data visualization for progress monitoring). Infographic visual flow diagrams were created and shared with SMHPs which were used along side the progress monitoring graphs to guide decisions on when/how to modify the DRC (e.g., level-up the target behavior, reduce the number of reminders, change the reward options). We also created a brief instructional video to train teachers, SMHPs, and parents to use the technology.

### Test Phase

The test phase included 6 schools/SMHPs (one SMHP per school) that completed CLS-R training. Two of the six SMHPs had previously completed in-person training in a prior version of CLS. These SMHPs were included in the test phase in order to evaluate remote training procedures from the perspective of those who had experience with the in-person training approach. Note that new training was needed for this group since several aspects of CLS-R were different from the prior version of CLS (e.g., consolidation of group content to one less parent session and consultation meeting, use of video tools, electronic DRC) and because their original training with CLS had been over 5 years prior and unlikely to have sustained effects over that period of time (Owens et al., 2014). To test whether results were affected by this prior experience, we evaluated all outcomes (usability, acceptability, fidelity, skills confidence, motivation and knowledge and student outcomes) separately for the previously trained SMHPs and found a similar pattern of results (e.g., similar usability, acceptability and fidelity ratings and direction of outcome effects) as for the newly previously trained SMHPs; therefore, results for the combined group are presented.

SMHPs led student recruitment, attended training workshops and weekly consultations, and delivered the CLS intervention which included eight 60-min parent and eight 45-min child group sessions, two 60-min group teacher meetings, and one or two (> 90% had two) parent–teacher–student meetings. Training occurred over approximately two and a half months.

## Student Participant Recruitment and Screening

Student participants (*n* = 5–6 per each school) were referred by school staff due to inattention and/or hyperactivity-impulsivity and related academic and/or social problems. Eligibility criteria were: (1) elevated ratings of ADHD symptoms (i.e., six or more inattention symptoms and/or six or more hyperactive-impulsive symptoms endorsed by either the parent or teacher on the Child Symptom Inventory (CSI) as occurring ‘often’ or ‘very often’), (2) cross-situational impairment (home and school) in at least one domain on the Impairment Rating Scale by both parent and teacher (score of 3 or greater), (3) a caretaker available to participate in treatment, (4) caretaker and child reads/speaks English and (5) a primary classroom teacher who agreed to participate in the classroom component. Children taking medication were eligible as long as their regimens were stable. Consent forms (SMHP, parent and teacher) and an assent form (child), approved by the institutional review board, were completed by SMHPs, parents, teachers, and children. At each time point, SMHPs were paid $50, parents were paid $20 and teachers were paid $40 for completing measures.

## Student Characteristics

Student participants included 36 children in grades 2–5 across 6 elementary schools. Of the child participants, 23(63.9%) were male, and 5(13.9%) were taking medications for ADHD. Racial and ethnic background was diverse: 15(41.6%) were White/Caucasian/not Hispanic/Latino, 4(11.1%) were Asian/Not Hispanic/Latino, 2(5.5%) were Black or African American/Hispanic/Latino, 1(2.8%) were American Indian/Alaska Native/Hispanic/Latino, 7(19.4%) indicated more than one race (3 Hispanic/Latino, 4 not Hispanic/Latino), and 3(8.3%) indicated other or unknown race (3 Hispanic/Latino). Across races, thirteen students (35.1%) were Hispanic or Latino. Twenty-five (69.4%) parents reported graduating from college. The sample was representative of the populations within their respective schools with the exception of underrepresentation of Asian families (35% in participating schools) (perhaps due to English language entry criteria) and overrepresentation of White families (24.7% in participating schools) and Hispanic/Latino families (15.8% in participating schools).

## Measures

The following measures were used in the Test phase.

### Usability

## Usability of Technology

The System Usability Scale (Brooke, 1996) was completed by SMHP and trainers for each CLS-R component. The SUS is a reliable, 10-item scale for assessing technology usability that has been widely used and adapted for various web-based applications and interventions (Lyon et al., 2021). Items are rated on a 5-point Likert scale, with final total scores ranging from 0 to 100 (high usability). Scores above 68 indicate above average usability. Scores above the cutoffs of 50.9, 71.4, 85.5 and 90 are considered “OK,” “Good,” “Excellent,” and “Best imaginable,” respectively (Bangor, 2009). The SUS has high internal consistency (α = 0.91) and high convergent validity with a separate rating of usability and user satisfaction (*r* = 0.81; (Bangor et al., 2008).

## Ease and Usefulness of CLS-R Features

SMHP and trainers rated the ease and usefulness of each CLS-R training feature (Zoom, screensharing, training videos, website materials/script, shared calendar, and progress monitoring) and parents/teachers rated ease and usefulness of the DRC on a 7 point scale (-3 = “very difficult/useless,” 0 = “neither easy/useful nor difficult/useless,” 3 = “very easy/useful”). Trainers also rated time spent setting up and troubleshooting technology. Optional open-ended questions asked about overall impressions with CLS-R, impressions about specific features/technology, and additions to the website or features they thought might be helpful.

### Acceptability

SMHPs provided ratings of overall quality of the program (1 = “very low” to 5 = “very high”), the CLS-R workshops (usefulness and engagement rated 1 = “poor” to 5 = “excellent”), whether they would recommend program to others (1 = “strongly not recommend to 5 = “strongly recommend”), and comfort level being observed/coached by trainers during parent, child and teacher meetings (1 = “very uncomfortable” to 7 = “very comfortable”). Trainers rated overall preference for remote vs. in-person training (1 = “definitely/very much prefer live” to 5 = “definitely/very much prefer remote”). Parents and teachers rated overall satisfaction (1 = “very dissatisfied” to 5 = ”very satisfied”), program appropriateness (1 = “very inappropriate” to 5 = “very appropriate”) and whether they would recommend the program to others (1 = “strongly not recommend” to 5 = “strongly recommend”).

### Implementation Fidelity

## Training Fidelity

Content fidelity assessed trainer adherence to essential training elements during workshops and consultation meetings. Fidelity measures were completed by trainers, and 57% were double-coded by independent observers for reliability (percent agreement = 99.9%). Trainers covered 99.4% of essential training elements during workshops and consultation meetings.

## SMHP Fidelity

SMHP adherence in each session was rated by trained observers with 37% double-coded by an independent observer to estimate interrater reliability (agreement = 98.8%). *SMHP Content Fidelity* checklists measured the adherence to required parent, student, and teacher session elements, rated on a 3-point scale (0 = not covered, 1 = partially covered, 2 = fully covered). *Use of Coaching:* Trainers recorded (yes/no) whether they provided content or prompted SMHPs to provide any session elements. *Quality Implementation* assessed SMHP’s EBP delivery factors (e.g., clearly provided content, asked questions to ensure participant understanding), group facilitation and management skills (e.g., maintained balance among group members, managed time effectively), and partnering skills (e.g., displayed warmth, conveyed enthusiasm) rated on a 5 point scale with higher scores indicating greater implementation quality.

## Session Attendance

SMHP, parent, student and teacher attendance at their respective group sessions and parent–teacher–student DRC meetings was monitored by the study team.

## DRC Implementation

The percentage of school days the DRC was implemented was based on the number of submitted DRC surveys out of the overall number of possible school days (excluding school holidays/events, child absences, and substitute teacher days) during the intervention.

### Effectiveness

## SMHP EBP confidence, motivation, and knowledge

SMHPs rated their confidence and motivation (1 = “not at all” to 5 = “extremely confident/motivated”), and knowledge (1 = “poor” to 5 = “excellent”) for implementing each EBP component (parent, child, teacher/parent–teacher collaboration) at baseline and post-treatment. Ratings were averaged separately for confidence, motivation, and knowledge for each component at each timepoint. Specific skills rated within each component were as follows: 1) *parent component*: explaining ABC model, labeled praise, home-based rewards, quality time, routines, homework planning, effective instructions, planned ignoring, response cost and stress management (αs = 0.81–0.92), 2) *child component:* labeled praise, effective instructions, differential reinforcement, response cost, attention checks and whole group intervention (αs = 0.65–0.69, 3) *teacher component* (αs = 0.75–0.89): school rewards, DRC, troubleshooting DRC, ABC model, behavior management, encouraging child skills, and collaborating on homework plans and school–home-based reward systems.

## Student Outcomes

These measures were completed at baseline and post-treatment:

*ADHD and ODD Symptoms*. Teachers and parents completed the *Child and Symptom Inventory* (CSI; Gadow, & Sprafkin, 2002). The ADHD and ODD items were rated on a 4-point scale from 0 = “never” to 3 = “very often.” The CSI has normative data and acceptable test–retest reliability and predictive validity for ADHD and ODD diagnoses (Sprafkin et al., 2002). Total ADHD and ODD scale scores were used to assess baseline and post-treatment symptom severity (αs = 0.90–94).

## Global Impairment

Global ratings of the severity of impairment were captured using the 7-point Clinical Global Impressions (CGI) Scale, Improvement (Busner & Targum, 2007) administered weekly and at post-treatment to parents (1 = “much improved” to 7 = “much worse”). Weekly ratings were plotted in graphs on the website for viewing by trainers, SMHPs, parents and teachers.

## Organizational Skills

Teachers and parents completed the Children’s Organizational Skills Scale (COSS; Abikoff & Gallagher, 2009). Items are rated on a 4-point scale (1 = “hardly ever or never” to 4 = “just about all the time”). The parent and teacher versions of the total organization problems score were used in the present study (αs = 0.92–96), with lower scores indicating better organizational functioning.

## Social-Behavioral Functioning

Parents and teachers completed the *prosocial behaviors* (αs = 0.78-0.80) and *peer problems* (αs = 0.50-0.72) subscales from the Strengths and Difficulties Questionnaire (SDQ; Goodman, 2001). Items were rated on a 3-point scale = “not true,” 1 = “somewhat true,” 2 = “certainly true”). The SDQ has good psychometric properties including adequate test–retest reliability (0.61-0.74) for parent and teacher versions.

## Homework Functioning

Parents completed the 20-item *Homework Problem Checklist* (Anesko et al., 1987). Items are rated on a 4-point scale (0 = “never” to 3 = “very often”). Higher scores on the measure indicate more severe homework problems. The total homework problems score was used in the present study (α = 0.94).

## Results

Descriptive statistics (means or medians, ranges or SDs) are reported separately for each stakeholder group for usability, acceptability and fidelity measures. Paired t-tests and effect sizes (Cohen’s *d*) are presented for SMHP and student outcomes.

## Usability

### SMHP-Reported Usability

**Fig. 1:**
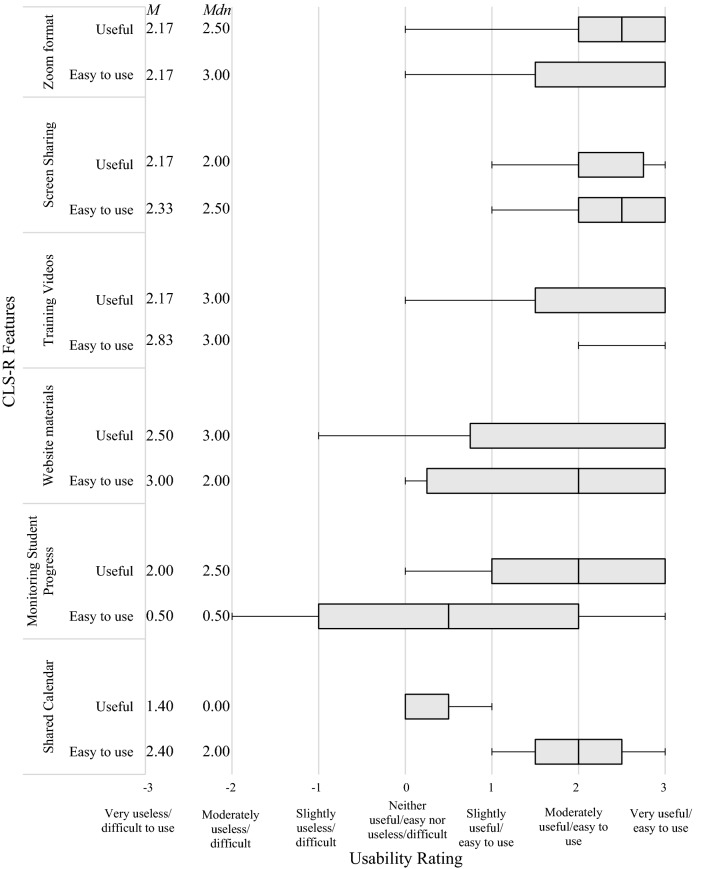
*Boxplot of school mental health providers’ usability ratings of specific CLS-R features. Note*. The median is represented as the center bar with the first and third quartiles plotted on the left and right, respectively. The plotted whiskers on the box plot represent the minimum and maximum scores. The mean (M) and median (Mdn) values for ratings of usefulness and ease of use for each component are reported to the left of the box plot

Overall ratings of CLS-R usability from the six SMHPs were above average (mean SUS = 87, “excellent”; range = 76 “good”-94 “best imaginable”). As shown in Fig. 1, ratings for ease and usefulness of the website and remote training features were generally high with means in the moderately to very easy/useful range. Ratings for the ease of using the tracking/monitoring features were slightly lower (mean between “neutral” to “slightly easy”) with some reporting difficulty toggling between graphs for multiple students. We also note that initially, SMHPs provided completed copies of DRCs to our team for electronic entry. However, usability and acceptability concerns from SMHPs prompted us to create an automated e-DRC tool; SMHP ratings of ease and usefulness subsequently increased (of note, this method also was deemed acceptable by teachers, see below). The shared calendar feature was rated as moderately easy to use on average, but less useful (mean between “neutral” to “slightly easy”) due to lack of integration with their other calendars. One SMHP commented the electronic scripts were “distracting,” “the screen [is] too small,” and “[scripts] took too long to load.”

### Trainer-Reported Usability

Trainer overall ratings of usability for CLS-R were above average (mean SUS = 88, “excellent”; range = 58 “OK” -98 “best imaginable”; 80% scored 90 or above “best imaginable”). All trainers rated zoom, training videos, and progress monitoring tools as very easy and very useful. All rated the electronic script as moderately to very easy/useful, and the parent demonstration videos as moderately to very easy/useful. One trainer provided overall ratings of moderately easy and useful for progress monitoring, but commented that it was tricky interpreting the graph when goals were switched during the treatment and that it was a bit cumbersome toggling between students. The shared calendar was rated by two trainers as slightly to moderately difficult to use because it was not integrated with their other calendars. Trainers reported spending an average of 5 min (range: 1–15 min) setting up the technology prior to sessions and an average of 1 min (range: 0–13 min) troubleshooting technology.

### Parent/Teacher-Reported Usability

Eighty-six percent of teachers reported that entering e-DRC points was moderately to very easy and useful, and they used this daily. Eighty-six percent of parents found that viewing the daily DRC points their child earned when sent via automated email was easy and useful and that they received these reports daily.

## Acceptability

### SMHP-Reported Satisfaction

All six SMHPs indicated they would “recommend” or “strongly recommend” the program, and the majority rated the overall program quality as “high” or “very high.” Remote training workshop usefulness was rated as “good” or “excellent” across all six SMHPs for all workshops. Ratings of engagement were also consistently “good” or “excellent” except for one workshop when one SMHP reported finding it “a little hard to go from rushing around my school job to stopping suddenly and shifting focus for a 2-h training while I was still onsite at my school. At times I felt distracted knowing what was happening onsite.” All SMHPs reported feeling comfortable being observed via Zoom for coaching during child and teacher sessions, and 5 of 6 reported feeling comfortable being observed for parent sessions. SMHPs were quite favorable about remote training logistics (e.g., per one SMHP “I loved this aspect of the program. So much simpler than driving across town to weekly trainings!”).

### Trainer Satisfaction

All trainers very much (N = 4) or mostly (N = 1) preferred the remote training over in-person training and commented that they favored the remote training due to time savings (approximately 1.5 h per session), convenient scheduling, and reduced travel cost.

## Parent- and Teacher-Reported Satisfaction

Over 90% of parents and teachers reported that they were satisfied or very satisfied with the program, rated the program as appropriate or very appropriate for attention/behavioral problems and would recommend or strongly recommend the program to others. All teachers and most parents (86% to 97% depending on type of session) reported that they were comfortable having the trainer observe the sessions remotely.

## Implementation Fidelity

### SMHP Program Adherence and Use of Live-Coaching

SMHPs fully or partially delivered 98.8% of program content elements on average. We also evaluated the percentage of sessions during which trainers provided any content (e.g., troubleshot parental concerns during homework review) or prompted clinicians to deliver content (e.g., reminded SMHPs to review omitted content while reviewing a handout). During CLS-R, trainers prompted SMHPs to provide content during 17.9% of sessions and provided content in only 7.7% of sessions. Implementation quality was high for all SMHPs (CLS-R *M* = 4.75, *SD* = 0.39).

### Parent, Student and Teacher Attendance

Attendance for parents at parent group averaged 76.9% (SD: 27.6, range: 11–100%); 86% attended at least half the sessions. Attendance for students at student groups averaged 91% (SD: 13.6, range: 37.5–100%); 97.2% attended at least half of all sessions. All teachers attended the two group meetings and DRC meetings (1 or 2 per student; > 90% held 2 meetings).

### DRC Implementation

Teachers completed the DRC an average of 96.6% of possible days (SD = 5.6%; range 80.6–100%). Teachers implemented the DRC an average 25.2 days (range 8 to 47) per student out of possible school days from when the DRC was started. Note that possible school days varied across students depending on when the DRC was started.

## Effectiveness

### SMHP-Reported EBP Skills Confidence, Motivation, and Knowledge

Table 3 presents results for SMHP EBP skill confidence, motivation, and knowledge at baseline and post-treatment for those receiving CLS-R. Paired t-tests and effect sizes (Cohen’s *d*) are presented for each comparison. SMHPs average ratings significantly improved from baseline to post-treatment for both perceived knowledge of skills and confidence in delivering skills across all program components (parent, child, and teacher). Ratings of motivation for teaching/coaching the skills across each of the components were moderate to high at baseline and did not show significant change at post-treatment.

**Table 3:** SMHP Ratings of Skill Confidence, Motivation, and Knowledge for those receiving CLS-R

Measure	Component	Baseline	Post	Cohen’s d^3^ (95% CI)	p-value
		*M (SD)*	*M (SD)*		
Confidence^1^	Parent	2.55 (.72)	3.89 (.60)	− 2.70 (− 4.49, − 0.88)	p = .001*
	Teacher	2.97 (.27)	3.83 (.65)	− 1.43 (− 2.41, − 0.40)	p = .005*
	Child	3.53 (.34)	4.11 (.39)	− 1.99 (− 3.40, − 0.53)	p= .005*
Motivation^1^	Parent	3.61 (.75)	3.77 (.77)	− 0.30 (− 1.10, 0.54)	**p = .502**
	Teacher	4.08 (.64)	4.00 (.72)	0.21 (− 0.50, 0.90)	**p = .575**
	Child	3.86 (.60)	4.11 (.44)	− 0.69 (− 1.57, 0.24)	**p = .151**
Knowledge^2^	Parent	2.70 (.66)	3.94 (.64)	− 2.43 (− 4.08, − 0.75)	p = .002*
	Teacher	3.00 (.37)	3.88 (.64)	− 1.37 (2.33, -0.36)	p = .006*
	Child	3.41 (.43)	4.28 (.48)	− 2.08 (− 3.54, − 0.58)	p= .004*

^1^Scale for confidence and motivation items: 1 = not at all, 2 = slightly, 3 = moderately, 4 = very, 5 = extremely confident/motivated

^2^Scale for knowledge items: 1 = poor, 2 = fair, 3 = good, 4 = very good, 5 = excellent

^3^For SMHP outcomes, *d* in a negative direction indicates improved confidence, motivation or knowledge from baseline to post-treatment and *d* in a positive direction indicates decreased confidence, motivation or knowledge from baseline to post-treatment

^*^Significant after within-domain Benjamini–Hochberg false discovery rate correction

### Student Outcomes

Table 4 presents results for parent and teacher ratings of ADHD and ODD symptoms and functional impairment at baseline and post-treatment for schools receiving CLS-R. Paired t-tests and effect sizes (Cohen’s *d*) are presented for each pre-post outcome measure. Models adjusted for school clustering yielded the same pattern of findings and therefore unadjusted models are presented with Bonferroni FDR correction for multiple comparisons. Results reveal significant reductions in ADHD and ODD symptom severity and significant improvement in global functioning from baseline to post-treatment with medium to large effects (*d*s = 0.45–1.28). Parent ratings also show significant reductions in homework and organization difficulties, and peer problems from baseline to post-treatment (*d*s = 0.52-0.99). Teacher ratings show significant improvement in prosocial skills (*d* = -0.41), but this did not survive Bonferroni FDR correction, and changes in organization (*d* = 0.34) were not significant.

**Table 4:** Means (SDs) for student outcome measures^*1*^

Measure	Informant	Baseline	Post	Cohen’s d^2^ (95% CI)	p-value
		*M (SD)*	*M (SD)*		
ADHD symptom severity	Parent	30.30 (10.16)	19.63 (6.34)	1.28 (0.76, 1.79)	p < =.001*
	Teacher	32.13 (9.57)	23.10 (10.44)	0.98 (0.57, 1.41)	p < =.001*
ODD symptom severity	Parent	9.30 (4.70)	6.70 (3.68)	0.76 (0.33, 1.19)	p < = .001*
	Teacher	8.55 (6.78)	6.29 (5.48)	0.45 (0.08, 0.82)	p < = .017*
Global improvements (CGI)	Parent	3.43 (1.17)	2.89 (1.20)	0.45 (0.05, 0.83)	p < = .026*
	Teacher	4.3 (1.15)	3.63 (1.16)	0.94 (0.50, 1.36)	p < = .001*
Organization problems	Parent	2.66 (0.39)	2.30 (0.41)	0.99 (0.54, 1.44)	p < = .001*
	Teacher	2.59 (0.33)	2.49 (0.38)	0.34 (− 0.03, 0.71)	p < = .073*
Prosocial behavior	Parent	8.00 (2.00)	8.31 (1.44)	− 0.22 (− 0.59, 0.15)	p < = .245*
	Teacher	5.73 (2.94)	6.63 (2.67)	− 0.41 (− 0.78, − 0.04)	p < = .032*
Peer problems	Parent	2.52 (1.94)	1.83 (1.58)	0.52 (0.12, 0.90)	p < = .010*
	Teacher	2.13 (2.37)	2.37 (2.08)	− 0.13 (− 0.48, 0.23)	p < = .491*
Homework problems	Parent	51.04 (13.6)	41.79 (12.69)	0.88 (0.44, 1.30)	p< = .001*

*ADHD * attention-deficit/hyperactivity disorder; *ODD* oppositional defiant disorder; *CGI * clinical global impression scale;

^*^Significant after within-domain Benjamini–Hochberg false discovery rate correction

^1^Data reported for parents (*n* = 29) and teachers (*n* = 31) who completed baseline and post-treatment measures prior to school closures

^2^For child symptom and problem domain outcomes *d* in a negative direction indicates improvement from baseline to post-treatment and in a positive direction indicates deterioration from baseline to post-treatment. For prosocial behavior, *d* in a negative direction indicates deterioration from baseline to post-treatment and a positive direction indicates improvement

## Discussion

Evidence-based behavioral treatments for ADHD are well-established, yet SMHPs are seldom trained in these approaches, limiting the extent to which these practices are implemented in school settings. Our goal was to develop an efficient, high-quality, acceptable web-based, remote training for an empirically supported school–home behavioral intervention as a first step toward increasing access, quality, and feasible utilization of evidence-based treatments among school mental health providers. Our quantitative and qualitative data from six SMHPs who received CLS-R training document that this approach provides an acceptable, usable, and effective alternative to in-person training from both SMHP and trainer perspectives with the benefit of greater feasibility, reduced time and cost. Furthermore, we observed little need for active coaching and high implementation quality during remote training, suggesting that remote training appears to allow for rapid implementation of newly learned skills. Our study also adds to existing literature on remote training by identifying/incorporating specific aspects which appear to enhance provider experience, such as enhanced training opportunities due to ease of access to training materials and trainers, as well as potential for co-learning with SMHPs at other school sites. Parent- and teacher-reported student outcomes reveal gains similar to those obtained from prior studies of in-person SMHP training (Pfiffner et al., 2011, 2016), providing further evidence for the promise of web-based, remote training for SMHPs.

Our translation of in-person to remote training was a collaborative process between SMHPs, trainers, our team of clinical researchers, and university-based web-developers who together co-developed the adapted the remote training protocol. Our key stakeholders, SMHPs and trainers, were involved throughout the discovery, design, build and test phases, which likely contributed to the favorable usability ratings from SMHPs and trainers alike regarding the key elements of CLS-R including the CLS-R website and videoconferencing tools. It is notable that these findings were obtained prior to the pandemic-related stay-at-home orders and school closures when Zoom videoconferencing became ubiquitous. For most trainers and SMHPs, this was their first time using Zoom. Many of our trainers and SMHPs commented that their experience with CLS-R prepared them well for the transition to fully remote telehealth sessions during the school closures. Qualitative feedback from SMHPs and trainers revealed that remote training allowed for more scheduling flexibility, time saving, and reduced travel costs and eliminated commuting challenges that were common during in-person training where workshops and consultation sessions typically required travel to either a district location or university space. They also appreciated the 24/7 access and ease of navigation to online program materials.

Overall acceptability of CLS-R was high. As we have found for in-person training in the past (Pfiffner et al., 2011, 2016), SMHPs were appreciative of the professional development, immediate feedback, and learning of new skills for parents and students afforded by the close supervision offered by the program. All of the SMHPs gave high ratings for the remotely conducted workshops and consultations, reporting that they were both useful and engaging. However, we also heard from SMHPs that there may be some drawbacks to remaining at their school site when trainings are conducted during school hours (e.g., distracted by ongoing school activities, interruptions from school staff) which would need to be managed. Acceptability was supported by trainers who unanimously preferred remote over in-person training and parents and teachers who were generally comfortable with the presence of a remote observer during sessions.

Importantly, the remote approach effectively trained clinicians to excellent levels of fidelity comparable to in-person training based on prior publications (parent sessions: *M* = 94%, child sessions: *M* = 97%) (Pfiffner et al., 2016), without sacrificing acceptability or utility. In fact, trainers used coaching prompts of content during only a few sessions and ratings of implementation quality were similar to those in prior studies of in-person training (parent sessions: *M* = 4.4/5; child sessions: *M* = 4.4/5) (Pfiffner et al., 2016). These findings suggest that we were successful at incorporating effective features of our in-person approach into the remote format (e.g., practice runs with materials and interactive activities). In contrast to other webinar trainings, our remote training sessions were small and interactive (e.g., role plays) and allowed for personalizing methods to meet the individual needs of SMHPs identified during meetings and in weekly feedback surveys. In addition, trainers supported one another during weekly trainer meetings where we reviewed training methods, website functionality, and engagement strategies for zoom. Together, these methods of ongoing support likely contributed to the high fidelity and ratings of satisfaction. SMHPs also reported improved and high rates of confidence and knowledge in explaining and coaching EBP skills with teachers, parents, and children from baseline to post-training. SMHP’s motivation to use these skills was high throughout receiving remote training during CLS-R. These findings are critical especially given the number of web-based training approaches used in recent years (Olson et al., 2021; Owens et al., 2019; Washburn et al., 2021). Further study of these remote training approaches to effectively prepare, engage, and motivate SMHPs during trainings may help to identify key mechanisms of implementation outcomes and further improve how we train trainers in the remote training approach.

The remote training of SMHPs was associated with high parent, student and teacher attendance at intervention sessions similar to prior studies of in-person training (parent: *M* = 79%, Student: *M* = 92%;) (Pfiffner et al., 2016). Interestingly, the automated DRC yielded a higher percentage of completed DRCs than we found previously using the paper–pencil version (*M* = 70%) (Meza et al., 2020). The vast majority of teachers and parents reported that the automated DRC process was at least moderately easy and useful. Greater compliance with and tracking of DRCs made possible by the automated approach likely contributed to these more favorable results. CLS-R yielded similar effects (i.e., most with effect sizes in moderate-large range) on student outcomes per parent and teacher report as found in prior studies of CLS with in-person training (Pfiffner et al., 2011) and a controlled comparison of CLS in-person training and usual care (Pfiffner et al., 2016). Although school closures precluded the planned larger scale comparison of in-person vs remote training, initial findings suggest that the clinician training format did not differentially impact student outcomes. We also replicated positive effects of this treatment on student outcomes across school and home settings, thereby further supporting this integrated treatment model implemented by SMHPs. These findings are in line with research showing that well-designed online trainings can be at least as effective as in-person methods (Becker & Jensen-Doss, 2013; Mullin et al., 2016; Olson et al., 2021).

## Limitations

Primary limitations are the small number of SMHPs and schools and reliance on open trials. As a result, we cannot rule out the possibility that SMHP and student outcomes were due to factors such as time, maturation, treatment expectancies, or nonspecific training effects rather than the CLS-R program. Findings may not generalize to SMHPs with different levels of EBT or remote learning experience or resources. Findings of similar SMHP fidelity, satisfaction and student outcomes from in-person training in other CLS trials increase our confidence in these results. Still, a randomized controlled trial with a larger sample testing the efficacy of CLS-R against CLS would more definitively determine whether the CLS-R effects are equivalent to in-person training. Unfortunately, due to disruptions from COVID-19 and prolonged school closures, we were not able to complete our RCT or measure long-term effects of the program, so while short-term effects are encouraging, we do not have information about whether the continued access to the website materials resulted in sustained use.

These findings, consistent with prior studies (Pfiffner et al., 2011, 2016), suggest that SMHPs can feasibly implement the program as part of their salaried workday. Although remote training increased feasibility due to time and cost savings and increased scheduling flexibility, the remote training included some training and supervision sessions of SMHPs after school hours for which they received stipends supported by grant funds. Future iterations of the training could incorporate professional leave time or protected time within the salaried workday for program training. Scalability also could be increased by increasing the size of the training groups. In addition, remote training required some district resources (i.e., laptops for SMHPs, Wifi). Because the software was developed with the university’s technology office using existing software packages available to researchers, there are currently no ongoing costs associated with the CLS-R website; however, it is important to note that most software requires ongoing data cloud storage and other costs for maintaining, which should be considered when estimating cost and savings.

Limitations of the platform we used complicated development of some features on our website (e.g., aspects of DRC functionality, calendar function). We note this as a general challenge in development of digital tools due to limits in personalization capabilities of web-platforms requiring building advanced software builds, and budgetary limitations often affecting progress from the design to build stage. Broad uptake and sustainability in digital methods will require continual focus on maximizing ease of use and usefulness over time for the users.

We also note that while racially and ethnically diverse, our sample was underrepresented for Asian American families, which reflects the larger disparity in ADHD treatment found among Asian Americans (Chung et al., 2019). Further research is warranted to examine linguistic and cultural adaptations as well as more effective strategies for treatment engagement to include a more representative sample of Asian students in future CLS programming (Lau, 2006).

## Conclusions

Overall, findings from this feasibility study support the promise of using web-based remote methods for training school clinicians. This approach appears to be a feasible training format with the potential for reducing costs and improving access to evidence-based mental health services. It is notable that remote training and telehealth approaches necessarily increased during the pandemic. We expect that these approaches will only gain in popularity after the pandemic in part due to ongoing improvements in technology and also due to the challenges associated with geographic barriers and cost of in-person training.

## References

[CR1] Abikoff H, Gallagher R (2009). Children’s Organizational Skills Scale. Journal of Psychoeducational Assessment.

[CR2] Anesko KM, Schoiock G, Ramirez R, Levine FM (1987). The Homework Problem Checklist: Assessing children’s homework difficulties. Behavioral Assessment.

[CR3] Bangor A (2009). Determining what individual SUS scores mean: Adding an adjective rating scale. Journal of Usability Studies..

[CR4] Bangor A, Kortum PT, Miller JT (2008). An empirical evaluation of the system usability scale. International Journal of Human-Computer Interaction.

[CR5] Becker EM, Jensen-Doss A (2014). Therapist attitudes towards computer-based trainings. Administration and Policy in Mental Health and Mental Health Services Research.

[CR6] Beidas RS, Edmunds JM, Marcus SC, Kendall PC (2012). Training and consultation to promote implementation of an empirically supported treatment: A randomized trial. Psychiatric Services.

[CR7] Brooke J (1996). SUS a quick and dirty usability scale. Usability Evaluation in Industry.

[CR8] Busner J, Targum SD (2007). Global impressions scale: Applying a research. Psychiatry (edgmont).

[CR9] Chung W, Jiang SF, Paksarian D, Nikolaidis A, Castellanos FX, Merikangas KR, Milham MP (2019). Trends in the prevalence and incidence of attention-deficit/hyperactivity disorder among adults and children of different racial and ethnic groups. JAMA Network Open.

[CR10] DuPaul, G. J., & Langberg, J. M. (2015). Educational impairments in children with ADHD, In RA Barkley (Ed), ADHD in Children and Adults: A Handbook for Diagnosis, Assessment, and Treatment (4th edition), NY: Guilford Press, pp 169–190.

[CR11] Evans SW, Owens JS, Wymbs BT, Ray AR (2018). Evidence-based psychosocial treatments for children and adolescents with attention deficit/hyperactivity disorder. Journal of Clinical Child and Adolescent Psychology.

[CR12] Fabiano GA, Pyle K (2019). Best practices in school mental health for attention-deficit/hyperactivity disorder: A framework for intervention. School Mental Health.

[CR13] Fabiano GA, Vujnovic RK, Pelham WE, Waschbusch DA, Massetti GM, Pariseau ME, Naylor J, Yu J, Robins M, Carnefix T (2010). Enhancing the effectiveness of special education programming for children with attention deficit hyperactivity disorder using a daily report card. School Psychology Review.

[CR14] Frank HE, Becker-Haimes EM, Kendall PC (2020). Therapist training in evidence-based interventions for mental health: A systematic review of training approaches and outcomes. Clinical Psychology: Science and Practice.

[CR15] Gadke DL, Kratochwill TR, Gettinger M (2021). Incorporating feasibility protocols in intervention research. Journal of School Psychology.

[CR16] Gadow, K. D., & Sprafkin, J. N. (2002). Child symptom inventory 4: Screening and norms manual. NY: Checkmate Plus.

[CR17] Goodman R (2001). Psychometric properties of the strengths and difficulties questionnaire. Journal of the American Academy of Child and Adolescent Psychiatry.

[CR18] Harrison JR, Vannest K, Davis J, Reynolds C (2012). Common problem behaviors of children and adolescents in general education classrooms in the united states. Journal of Emotional and Behavioral Disorders.

[CR19] Herschell AD, Kolko DJ, Baumann BL, Davis AC (2010). The role of therapist training in the implementation of psychosocial treatments: A review and critique with recommendations. Clinical Psychology Review.

[CR20] Johnston C, Mash EJ (2001). Families of children with attention-deficit/hyperactivity disorder: Review and recommendations for future research. Clinical Child and Family Psychology Review.

[CR21] Kaiser NM, McBurnett K, Pfiffner LJ (2011). Child ADHD severity and positive and negative parenting as predictors of child social functioning: Evaluation of three theoretical models. Journal of Attention Disorders.

[CR22] Khanna MS, Kendall PC (2015). Bringing technology to training: web-based therapist training to promote the development of competent cognitive-behavioral therapists. Cognitive and Behavioral Practice.

[CR23] Kobak KA, Craske MG, Rose RD, Wolitsky-Taylor K (2013). Web-based therapist training on cognitive behavior therapy for anxiety disorders: A pilot study. In Psychotherapy American Psychological Association.

[CR24] Kobak KA, Wolitzky-Taylor K, Craske MG, Rose RD (2017). Therapist training on cognitive behavior therapy for anxiety disorders using internet-based technologies. Cognitive Therapy and Research.

[CR25] Kuriyan AB, Pelham WE, Molina BSG, Waschbusch DA, Gnagy EM, Sibley MH, Babinski DE, Walther C, Cheong J, Yu J, Kent KM (2013). Young adult educational and vocational outcomes of children diagnosed with ADHD. Journal of Abnormal Child Psychology.

[CR26] Lau AS (2006). Making the case for selective and directed cultural adaptations of evidence-based treatments: Examples from parent training. Clinical Psychology: Science and Practice.

[CR27] Lyon AR, Munson SA, Renn BN, Atkins DC, Pullmann MD, Friedman E, Areán PA (2019). Use of human-centered design to improve implementation of evidence-based psychotherapies in low-resource communities: Protocol for studies applying a framework to assess usability. JMIR Research Protocols.

[CR28] Lyon AR, Pullmann MD, Jacobson J, Osterhage K, Al Achkar M, Renn BN, Munson SA, Areán PA (2021). Assessing the usability of complex psychosocial interventions: The intervention usability scale. Implementation Research and Practice.

[CR29] Lyon AR, Stirman SW, Kerns SEU, Bruns EJ (2011). Developing the mental health workforce: Review and application of training approaches from multiple disciplines. Administration and Policy in Mental Health and Mental Health Services Research.

[CR30] Meza JI, Friedman LM, Dvorsky MR, Kass P, Chabbra D, Pfiffner LJ (2020). Outcomes of school-home intervention for attention and behavior problems: Teacher adherence matters. School Mental Health.

[CR31] Mikami AY (2010). The importance of friendship for youth with attention-deficit/hyperactivity disorder. Clinical Child and Family Psychology Review.

[CR32] Mullin DJ, Saver B, Savageau JA, Forsberg L, Forsberg L (2016). Evaluation of online and in-person motivational interviewing training for healthcare providers. In Families, Systems, & Health Families, Systems & Health Inc.

[CR33] Olson JR, Lucy M, Kellogg MA, Schmitz K, Berntson T, Stuber J, Bruns EJ (2021). What happens when training goes virtual? Adapting training and technical assistance for the school mental health workforce in response to COVID-19. School Mental Health.

[CR34] Ortiz C, Vidair HB, Acri M, Chacko A, Kobak K (2020). Pilot study of an online parent-training course for disruptive behavior with live remote coaching for practitioners. Professional Psychology: Research and Practice.

[CR35] Owens JS, Lyon AR, Brandt NE, Masia Warner C, Nadeem E, Spiel C, Wagner M (2014). Implementation science in school mental health: key constructs in a developing research agenda. In School Mental Health.

[CR36] Owens JS, McLennan JD, Hustus CL, Haines-Saah R, Mitchell S, Mixon CS, Troutman A (2019). Leveraging technology to facilitate teachers use of a targeted classroom intervention: Evaluation of the daily report card. Online (DRC.O) System, in School Mental Health.

[CR37] Pfiffner, L. J. (2011). All About ADHD: The Complete Practical Guide for Classroom Teachers (2nd ed.). Scholastic Inc.

[CR38] Pfiffner LJ, Kaiser NM, Burner C, Zalecki C, Rooney M, Setty P, McBurnett K (2011). From clinic to school: Translating a collaborative school-home behavioral intervention for ADHD. School Mental Health.

[CR39] Pfiffner LJ, McBurnett K (1997). Social skills training with parent generalization: Treatment effects for children with attention deficit disorder. Journal of Consulting and Clinical Psychology.

[CR40] Pfiffner LJ, Rooney ME, Jiang Y, Haack LM, Beaulieu A, McBurnett K (2018). Sustained effects of collaborative school-home intervention for attention-deficit/hyperactivity disorder symptoms and impairment. Journal of the American Academy of Child and Adolescent Psychiatry.

[CR41] Pfiffner LJ, Rooney M, Haack L, Villodas M, Delucchi K, McBurnett K (2016). A randomized controlled trial of a school-implemented school-home intervention for attention-deficit/hyperactivity disorder symptoms and impairment. Journal of the American Academy of Child and Adolescent Psychiatry.

[CR42] Proctor E, Silmere H, Raghavan R, Hovmand P, Aarons G, Bunger A, Griffey R, Hensley M (2011). Outcomes for implementation research: Conceptual distinctions, measurement challenges, and research agenda. Administration and Policy in Mental Health and Mental Health Services Research.

[CR43] Rakovshik SG, McManus F, Vazquez-Montes M, Muse K, Ougrin D (2016). Is supervision necessary? Examining the effects of internet-based CBT training with and without supervision. Journal of Consulting and Clinical Psychology.

[CR44] Robb JA, Sibley MH, Pelham WE, Foster EM, Molina BSG, Gnagy EM, Kuriyan AB (2011). The estimated annual cost of ADHD to the US education system. School Mental Health.

[CR45] Salesforce. (2018). Saleforce Community Cloud. https://salesforce.com

[CR46] Spiel CF, Evans SW, Langberg JM (2014). Evaluating the content of Individualized Education Programs and 504 Plans of young adolescents with attention deficit/hyperactivity disorder. In School Psychology Quarterly.

[CR47] Sprafkin J, Gadow KD, Salisbury H, Schneider J, Loney J (2002). Further evidence of reliability and validity of the child symptom inventory-4: parent checklist in clinically referred boys. In Journal of Clinical Child and Adolescent Psychology.

[CR48] Stewart RE, Stirman SW, Chambless DL (2012). A qualitative investigation of practicing psychologists attitudes toward research-informed practice: Implications for dissemination strategies. In Professional Psychology: Research and Practice.

[CR49] Torous J, Jän Myrick K, Rauseo-Ricupero N, Firth J (2020). Digital Mental Health and COVID-19: Using technology today to accelerate the curve on access and quality tomorrow. In JMIR Mental Health.

[CR50] Washburn M, Zhou S, Sampson M, Palmer A (2021). A Pilot study of peer-to-peer SBIRT simulation as a clinical telehealth training tool during COVID-19. In Clinical Social Work Journal.

